# A 3D Fully Convolutional Neural Network With Top-Down Attention-Guided Refinement for Accurate and Robust Automatic Segmentation of Amygdala and Its Subnuclei

**DOI:** 10.3389/fnins.2020.00260

**Published:** 2020-05-21

**Authors:** Yilin Liu, Brendon M. Nacewicz, Gengyan Zhao, Nagesh Adluru, Gregory R. Kirk, Peter A. Ferrazzano, Martin A. Styner, Andrew L. Alexander

**Affiliations:** ^1^Waisman Brain Imaging Laboratory, University of Wisconsin-Madison, Madison, WI, United States; ^2^Department of Psychiatry, University of Wisconsin-Madison, Madison, WI, United States; ^3^Department of Medical Physics, University of Wisconsin-Madison, Madison, WI, United States; ^4^Department of Pediatrics, University of Wisconsin-Madison, Madison, WI, United States; ^5^Department of Psychiatry, University of North Carolina-Chapel Hill, Chapel Hill, NC, United States; ^6^Department of Computer Science, University of North Carolina-Chapel Hill, Chapel Hill, NC, United States

**Keywords:** deep learning, fully convolutional neural network, amygdala, structural MRI, segmentation, harmonization, generalization

## Abstract

Recent advances in deep learning have improved the segmentation accuracy of subcortical brain structures, which would be useful in neuroimaging studies of many neurological disorders. However, most existing deep learning based approaches in neuroimaging do not investigate the specific difficulties that exist in segmenting extremely small but important brain regions such as the subnuclei of the amygdala. To tackle this challenging task, we developed a dual-branch dilated residual 3D fully convolutional network with parallel convolutions to extract more global context and alleviate the class imbalance issue by maintaining a small receptive field that is just the size of the regions of interest (ROIs). We also conduct multi-scale feature fusion in both parallel and series to compensate the potential information loss during convolutions, which has been shown to be important for small objects. The serial feature fusion enabled by residual connections is further enhanced by a proposed top-down attention-guided refinement unit, where the high-resolution low-level spatial details are selectively integrated to complement the high-level but coarse semantic information, enriching the final feature representations. As a result, the segmentations resulting from our method are more accurate both volumetrically and morphologically, compared with other deep learning based approaches. To the best of our knowledge, this work is the first deep learning-based approach that targets the subregions of the amygdala. We also demonstrated the feasibility of using a cycle-consistent generative adversarial network (CycleGAN) to harmonize multi-site MRI data, and show that our method generalizes well to challenging traumatic brain injury (TBI) datasets collected from multiple centers. This appears to be a promising strategy for image segmentation for multiple site studies and increased morphological variability from significant brain pathology.

## 1. Introduction

The amygdala is a key regulator of emotional arousal and is thought to regulate generalization or habituation of fear responses in normal and abnormal development (Adolphs et al., 2005; Knight et al., 2005; Öhman, 2005). Animal models have been used to differentiate subregions of the amygdala, identifying structural bases of fear generalization in basal and lateral nuclei distinct from output projections from centromedial regions (Amaral et al., 1992; LeDoux, 2007; Hrybouski et al., 2016; Kwapis et al., 2017), and reliable quantification of these substructures would be extremely useful. Accurate segmentation of the amygdala and specific subregions for quantitative analyses may provide better insights into fear and emotion processing and the role of the amygdala in traumatic brain injury and neuropsychiatric diseases. However, as a deep heterogeneous cluster of subregions, surrounded by vasculature, it remains an extremely difficult region to quantify. Compared with conventional automated software (Freesurfer, FSL), hand drawn amygdala boundaries can better capture cumulative contributions of biological and environmental stress, including autistic social impairment, physical abuse, institutional neglect and poverty (Nacewicz et al., 2006; Hanson et al., 2015). However, manual segmentation is extremely time-consuming and is prone to biases (Maltbie et al., 2012), highlighting the need for highly accurate automated segmentation methods. Currently, there are no reliable segmentation tools for subnuclei regions of the amygdala. Furthermore, the effects of image and subject variability from scanner, protocol and brain pathology on amygdala segmentation have not been previously investigated.

Segmentation methods for the amygdala can largely be classified into atlas-based and learning-based categories. A high resolution MRI atlas of the amygdala with defined subregions was recently described (Tyszka and Pauli, 2016); however, the utilization of this atlas to individual brain images is limited by the ability to anatomically spatially align the atlas. A promising strategy is the multi-atlas based method in which the segmentation of a target image is estimated by aligning it with one or more labeled atlases through registration (Babalola et al., 2009; Leung et al., 2010; Hanson et al., 2012). There is, however, a considerable computational cost associated with multi-atlas approaches since all of the atlases need to be deformably registered to each target image case using non-linear deformable transformations (Hanson et al., 2012). Additionally, the segmentation quality in multi-atlas approaches highly depends on the selection of the atlases and the fusion algorithm (Rohlfing et al., 2004; Aljabar et al., 2009). Other automatic population atlas-based segmentation packages are FreeSurfer and FSL, but overall their segmentation performances remain not optimal (Morey et al., 2009; Schoemaker et al., 2016) due to insensitivity to biologically-relevant variance (Hanson et al., 2015) and failure to capture subtle boundaries of centromedial nuclei when applied to single subjects (Saygin et al., 2017). Furthermore, neither Freesufer nor FSL support the segmentation of the subregions of the amygdala.Therefore, neither Freesurfer nor FSL performance are evaluated in this paper. A significant limitation with existing tools and prior work in this domain is that the effects of variability across scanners and protocols have not been investigated, nor have the effects of brain injuries on amygdala segmentation.

Recently, convolutional neural networks (CNN) have brought tremendous improvements in various computer vision tasks such as image classification and segmentation (Krizhevsky et al., 2012; Simonyan and Zisserman, 2014; He et al., 2016). Unlike traditional machine learning, CNN as a learning based approach can autonomously learn representations of data with increasing levels of abstraction via multiple convolutional layers without feature engineering. In CNNs, weights are shared and locally connected among convolutional layers, which significantly reduces the number of parameters compared with fully connected layers, making CNNs especially suitable for imaging tasks. Naturally, CNNs have been gradually becoming the tool of choice for medical imaging tasks. In medical image segmentation, a classification network was previously proposed using a sliding window scheme to predict the class probability of the center pixels of over-lapping patches (Ciresan et al., 2011). Since such a classification makes predictions for a single pixel at a time, this approach suffers from redundant computations and does not benefit from correlations across pixels. Long et al. (2015) first proposed then fully convolutional neural networks (FCNN) in which the fully connected layers are replaced with 1x1 convolution so that the network consists of convolutional layers only. This strategy allows dense predictions for multiple pixels in a single forward pass, and eliminates the limitation posed by fully connected layers on the size of the input image size. FCNN therefore serves as an effective general purpose engine for tasks of semantic image segmentation.

A widely-used FCNN architecture is “encoder-decoder,” which are popularized by U-Net (Ronneberger et al., 2015), 3D U-Net (Çiçek et al., 2016), V-Net (Milletari et al., 2016), and SegNet (Badrinarayanan et al., 2017). The encoder part compresses the input images into lower-resolution feature maps via downsampling or pooling layers, and the decoder part aims to recover the full-resolution label map from these feature maps for pixel-to-pixel semantic classification. These networks have similar encoders—a VGG-like (Simonyan and Zisserman, 2014) architecture is typically adopted, while they vary with respect to their decoder strategies. Multiple up-sampling strategies have been proposed for decoders, including deconvolution (Noh et al., 2015), bilinear upsampling and unpooling (Badrinarayanan et al., 2017). However, such design could pose a few problems when segmenting structures with small spatial extent. First, although consecutive strided convolutions or pooling operations employed in these networks enable a large receptive field, fine details may be lost and are difficult to remedy via simple non-learnable upsampling strategies or skip connections. For example, if a network has a downsample rate of 1/8 (as it employs three max-pooling layers with 2 × 2 filters with stride 2), an object with less than 8 voxels (such as the amygdala's subregions) in each dimension may not be well recovered later. Second, since down-sampling operations typically lead to great dimension reduction, the input images of these networks need to be large enough so as to preserve sufficient dimension after the compression of the encoder, for being further processed by the decoder. But larger image patches are more likely to be dominated by background voxels compared with smaller ones, leading to severe class imbalance problem. This makes the predictions more favorable to the background, which is particularly of concern for small objects. Although a weighted cross entropy loss function has been suggested to alleviate this problem (Ronneberger et al., 2015; Çiçek et al., 2016), choosing a proper weight map for all the classes is non-trivial. Another solution could be the Dice loss function (Milletari et al., 2016) which avoids tuning any extra hyperparameter and weighs false negatives and false positives equally. Hence, although these networks have plenty of success in segmentation tasks of large structures such as brain extraction (Zhao et al., 2018), lung (Negahdar et al., 2018), and breast segmentation (Dalmış, 2017), specific strategies for small structures are necessary.

Compared with larger structures, smaller ones like the amygdala and its subregions provide fewer signals to exploit, which makes the learning of discriminative features more challenging. Hu and Ramanan (2017) suggested that modeling context is particularly helpful for CNNs to recognize small objects, based on a key observation that humans can only accurately classify small faces with evidence beyond the object itself. In general, context can provide knowledge of a structure with respect to its surroundings and disambiguate objects with similar local visual appearances. Thus, incorporating context can critically improve recognition accuracy (Galleguillos and Belongie, 2010). In medical imaging, many studies have explored the idea of using input patches with various sizes for modeling multi-scale contextual information (de Brebisson and Montana, 2015; Moeskops et al., 2016; Ghafoorian et al., 2017; Kamnitsas et al., 2017). Most of these networks are organized in a multi-branch manner, where each branch independently processes patches of a certain type. In other patch-based CNN approaches, explicit spatial features obtained from a structural probabilistic atlas are combined with CNN features to provide additional spatial information (Kushibar et al., 2018). Another line of efforts focuses on enlarging kernels via dilated convolutions to integrate larger contextual information (Chen et al., 2018). Segmenting small structures with high accuracy is therefore reduced to the problem of finding the optimal trade-off between capturing sufficiently large context and retaining fine details, while alleviating the imbalanced class issue.

In light of the limitations of previous works, we present a dual-branch dilated residual FCNN with two parallel convolutions to extract both local context for alleviating the class imbalance issue and more global context. Residual connections (He et al., 2016) are added to facilitate the gradient flow and more importantly, feature reuse from earlier layers. In order to enhance such feature fusion, we additionally develop a top-down attention-guided (AG) refinement unit resided on residual connections to select useful low-level details from earlier layers to better complement the highly semantic feature maps from deep layers, which we believe can benefit the segmentation of small regions like the amygdala and subnuclei on structural T1-weighted images. In general, attention mechanisms can emphasize important features and suppress the irrelevant ones, mimicking human visual system, which has been broadly applied to various vision and natural language processing tasks (Bahdanau et al., 2014). A popular attention mechanism, “Squeeze & Excitation” (SE) module (Hu et al., 2018) which recalibrates channels by modeling channel interdependencies, has been shown to be effective in some medical images segmentation tasks (Roy et al., 2018). Different from SE, we utilize higher-level information as priors to recalibrate lower-level channels.

This study focused on two critical areas of brain image segmentation—(1) the parcellation of very small structures like the subnuclei of the amygdala, and (2) the application of whole amygdala segmentation across multiple scanners and variable brain injuries. For the parcellation of amygdala subnuclei, we evaluated the accuracy of our segmentation method by comparing it to other automated methods including two deep learning based and a multi-atlas based method. A preliminary version of the presented work appeared in Liu et al. (2018). We further demonstrate the benefits of the dual-branch design by analyzing the influence of each branch on final performance and compare the two design choices of our attention-guided refinement unit to SE module (Hu et al., 2018), showing that the top-down AG refinement unit is more suitable than SE in this application, and potentially in segmentation tasks of other small structures. Finally, we investigated a strategy to generalize the FCNN amygdala segmentation approach to a challenging Traumatic Brain Injury (TBI) dataset collected from multiple sites, despite the variability of contrast and image sensitivity across MRI scanner hardware (RF coils, in particular) and software (pulse sequences and protocols) and increased image heterogeneity associated with pathology, demonstrating its robustness to real-world practice.

## 2. Materials and Methods

### 2.1. Dataset

T1-weighted MRI data from 14 subjects (age mean (standard deviation) 28.9 years (6.5 years); range 18.5–43.4 years), each imaged in both morning and evening sessions on 2 days separated by 1 week (four total imaging sessions) on a GE MR750 3.0 T MRI scanner with the product 8-channel head coil. All participants provided written consent or assent as part of a procedure approved by the Human Subjects Institutional Review Board of the University of Wisconsin School of Medicine and Public Health. A whole-brain 3D inversion-recovery prepared fast gradient-echo T1- weighted sequence (inversion time TI = 600 ms; fast gradient echo readout TR/TE = 9.4/3.1 ms; 256 × 192 matrix, resampled to 256 × 256, over 240 mm field of view with 128 slices 1-mm thick) was prescribed as axial oblique slices angled so that the midpoint and splenium of the corpus callosum occupied the same plane (Nacewicz et al., 2012).

An iterative pre-processing pipeline used 3DSkullStrip (AFNI) (Cox, 1996) to output a roughly skull-stripped image, which was then coregistered to the MNI152 template by affine transform in FLIRT (FSL) (Jenkinson et al., 2012), and tissue priors reverse warped to native space for segmentation-based bias-field correction in FAST (FSL), the dilated bias field was applied to the original image, which was then more effectively skull-stripped, contrast-adjusted and squared to exaggerate gray matter-CSF differences, re-coregistered to the MNI template for better alignment of tissue priors and a final bias correction with FAST. This method was developed to preserve tissue in the lateral nucleus of the amygdala, which is otherwise frequently misclassified as CSF and erroneously darkened by bias corrections. The resultant images from each of the 4 sessions were coregistered in FLIRT to an individual-subject averaged space (1 mm isotropic) representing an affine transform equidistant to all 4 session images and averaged (Nacewicz et al., 2012), followed by landmark-based AC-PC alignment with concomitant cropping to 191 × 236 × 171 (Cox, 1996), and rotation to the “pathological plane” to match post-mortem atlases (Nacewicz et al., 2006).

**Figure 1:**
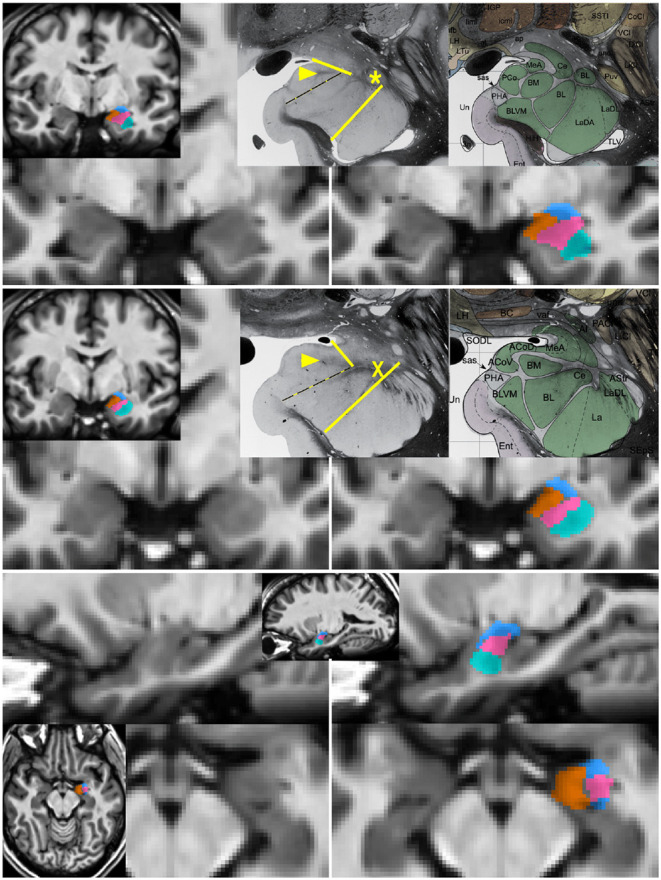
Segmentation of subnuclear groups by landmarks visible on single subject images. Unlabeled (left) and labeled (right) images at more posterior (top) and anterior (middle) coronal sections with representative histology and subdivisions from Mai et al. (3rd ed) (Mai et al., 2015). Tracing began in the coronal section with the “dogleg” of the basolateral nucleus (asterisk). The lateral nucleus (teal) was easily identifiable by the lower T1 intensity lateral to a linear border with the basolateral nucleus. The combined basal nuclei (pink) was defined starting in the plane of the dogleg, with the dorsal boundary following the thin white matter angling inferomedially along the central nucleus. The medial boundary of the basal group extends up to but not including the white matter encircling the cortical nucleus. A key landmark anterior to the dogleg is a spider-like white matter formation (middle, X) dividing all subdivisions and discernible in all single-subject images. When the white matter of the cortical nucleus was not visible, a spot of white matter at the triple junction with the medial nucleus (arrowhead in top and middle) or the most medial tip of white matter between basolateral and central nucleus was connected with the most medial extent of the subventricular/uncal white matter (dotted line). The cortico-superficial grouping (orange) extends superiorly to a line from the triple junction in posterior sections or the tip of white matter above basolateral nucleus on anterior sections to the more superolateral of the endorhinal sulcus or optic tract. The centromedial group (blue), includes all darker tissue above these boundaries. All nuclei were then refined to achieve smooth agreement in sagittal and axial views (bottom).

Both the left and the right amygdala were manually divided on the 4-session averaged 1 mm isotropic T1-weighted images into four subnuclear groups on each side—lateral, basal, cortico-superficial (olfactory) and centromedial subregions—by an amygdala anatomy expert (BN) based on visible landmarks largely matching those described by Amaral et al. (1992). Details of how subregions are defined are provided in Figure 1. We note that the slight in-plane downsampling to 1mm and the spatial normalization did not impair the manual labeling. Specifically, in the coronal plane the *lateral* nucleus was easily isolated due to its darker intensity. The combined *basal* nuclei went from the thin white matter capsule of the cortical nucleus medially to the intense, linear lateral border formed by the fibers passing through the plane along this edge and with careful effort to include the magnocellular “dogleg” portion and its white matter capsule; and the dorsomedial boundary of the basomedial region was formed by a straight line from the most ventromedial extent of the visible white matter around the “dogleg” down to the most ventromedial tip of the amygdala clearly visible above the hippocampal head. The combined *cortico-superficial* nuclei included all tissue bordering on the ambient cistern above the semiannular sulcus ventrally up to the more lateral of either the rhinal sulcus or lateral extent of the optic tract, with the lateral boundary defined by the white matter capsule of the cortical nucleus or the straight line boundary described above for the basal group. The combined *centromedial* group was bounded by white matter dorsally including a thin boundary between the central nucleus and putamen, extended ventromedially along the white matter forming the dorsomedial boundary of the “dogleg” of the basolateral nucleus, then a straight line extended dorsomedially to the more lateral of the rhinal sulcus or optic tract. The manual labeling of 10 ROIs per individual on 14 brains with two blinded repeats (four amygdalae) yielded intra-rater Dice overlap coefficients: Lateral = 0.89, Basal = 0.82, Centromedial = 0.77, Superficial = 0.75 and total amygdala using our previously published technique yielded excellent agreement (dice = 0.94). Manual tracing is, however, quite tedious and time-intensive, requiring 10–20 person-hours per brain, which limits application to larger data sets. Overall, the right and the left amygdala jointly account for about 0.05% of the whole brain volume of a single subject. Training and evaluation of the segmentation methods as described below were performed on single session (non-averaged) data using the segmentation labeling from the averaged data.

### 2.2. Network Backbone

**Figure 2:**
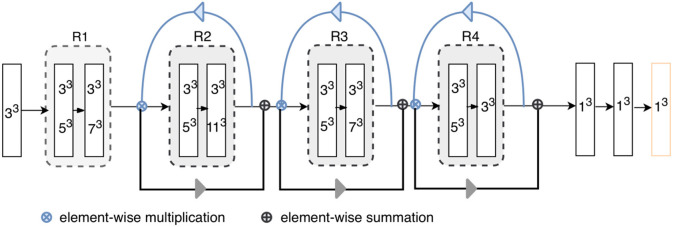
Architecture of the proposed model. “RX”s represent residual blocks (the residual connections are omitted here). The rectangles with two kernel sizes represent parallel convolutions, as illustrated in Figure 3. The attention weights generated using higher-level feature priors, denoted as blue arrows, are multiplied with the lower-level channels; then, the reweighted lower-level features are used to refine the next layers, as shown by gray arrow. Each layer except for the final classification layer (orange) is followed by batch normalization and ReLU.

**Figure 3:**
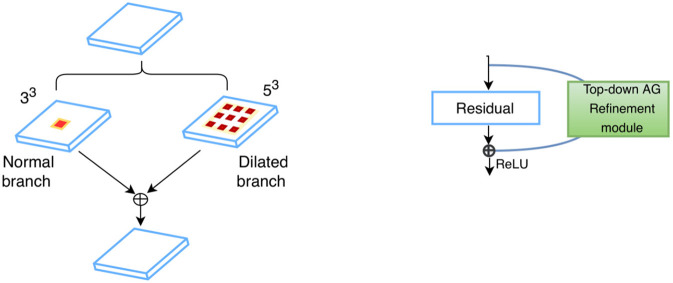
Feature fusion in parallel **(Left)** and series **(Right)**: kernels of two different sizes are applied in parallel, and the resultant feature maps are fused via element-wise summation; standard residual connections are adopted for serial feature fusion, where features from earlier layers are incorporated into deeper layers.

To incorporate larger contexts while alleviating class imbalance, we present a dual-branch model design (Figure 2), with one specializing in capturing multi-scale contexts and the other maintaining a small receptive field which helps the model focus on the ROIs. For any given feature map *U* ∈ ℝ^*H* × *W* × *D*^^,^ kernels of two different sizes are applied in parallel to perform two transformations $\Omega :U\to \hat{F}\in {\mathbb{R}}^{H^{\prime }\times W^{\prime }\times D^{\prime }}$ and $\psi :U\to \tilde{F}\in {\mathbb{R}}^{H^{\prime }\times W^{\prime }\times D^{\prime }}$, forming two branches. In order to more efficiently preserve information, dilated convolutions (Yu and Koltun, 2015) in place of down-sampling layers are adopted throughout the network, i.e., kernels are up-sampled with zeros inserted between weights so that the receptive field of the kernels can be expanded without incurring extra computational costs. The gap between elements in a kernel is *D*_*k*_−1, where *D*_*k*_ denotes the dilation rate, with standard convolution as a special case when *D*_*k*_ is 1. Therefore, the two branches are composed of 3^3^ kernels with *D*_*k*_1__ ≥ 1 (dilated branch) and *D*_*k*_2__ = 1 (standard branch), respectively. For example, a 5^3^ kernel for the dilated branch is a 3^3^ kernel with *D*_*k*_ = 2. Batch Normalization (Ioffe and Szegedy, 2015) and ReLU non-linearity (Glorot et al., 2011) are applied in sequence after convolutions. Information from both branches are then fused via element-wise summations before being fed into the next layer (Figure 3, left):

$$\begin{array}{l} F^{l}={\hat{F}}_{dilated}^{l}+{\tilde{F}}_{normal}^{l}, \end{array}$$

where *F*^*l*^ denotes the fused feature maps (FMs) for each layer *l*. The small dilation rates designed for standard branch are to ensure that it has a small receptive field of size 19 × 19 × 19 which can just enclose the whole amygdala. This allows for a detailed analysis of the ROIs and alleviates the class imbalance problem, since the receptive field determines the number of voxels that can influence model predictions per optimization step. For the dilated branch, the dilation rates are empirically set to be *D*_*k*_1__ = {1, 2, 4, 2, 8, 2, 4, 2, 1}, resulting in a receptive field of size 53 × 53 × 53, which can capture large contexts. The number of kernels for each branch is as follows: 30, 30, 40, 40, 40, 40, 50, 50, 50. In addition to such parallel feature fusion, residual connections (He et al., 2016) are also integrated into the network mainly for feature reuse (Chen et al., 2017) in series, which adds the features from a lower layer to those from a higher layer via skip connections (Figure 3, right). Both the parallel and serial feature fusion are shown in Figure 3. They are further enhanced by a top-down attention mechanism described in section 2.3.

### 2.3. Top-Down Attention-Guided Refinement Unit

**Figure 4:**
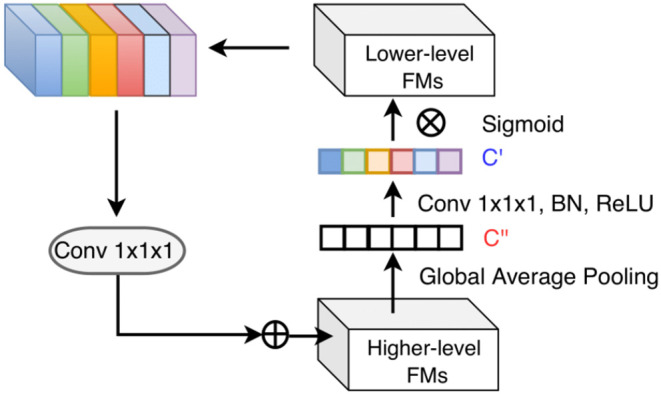
Top-down attention-guided refinement unit on residual connections, where lower-level features are recalibrated by higher-level information and incorporated into deeper layers. “FMs” denotes as feature maps. Channel-wise statistics of higher-level information are first extracted by global average pooling, and the interdependencies among channels are modeled by a 1 × 1 × 1 convolution followed by the sigmoid activation. The reweighted lower-level features are then added to the higher-level features.

CNNs are known to have an inherent feature hierarchy, where layers that are close to the inputs extract high-resolution spatial details and deeper layers form highly semantic but coarser features. A number of deep learning studies have explored to fuse multi-level features from different layers to enrich the feature representation (Hariharan et al., 2015; Long et al., 2015; Ronneberger et al., 2015; Lin et al., 2017; Zhang et al., 2018). Especially, segmentation of small objects is found to benefit from such feature reuse from earlier layers where fine-grained low-level details are abundant (Shrivastava et al., 2016; Lin et al., 2017). Nevertheless, indiscriminately fusing the different levels of features may not always be effective due to the semantic dissimilarity empirically found by Zhang et al. (2018). Motivated by their observation, we propose a top-down attention-guided refinement unit based on residual connections to supplement the typical feed-forward, bottom-up CNN, where the abundant semantic information from the higher layers can highlight and select the low-level details from lower layers, as shown in Figure 4. Given a set of features maps from earlier layers $F_{low}\in {\mathbb{R}}^{C^{\prime }\times H\times W\times D}$, a set from higher layers $F_{high}\in {\mathbb{R}}^{C^{\prime \prime }\times H\times W\times D}$, and the attention coefficients α ∈ ℝ^1 × 1 × 1 × *C*^′ the *refined* feature maps from higher layers can be defined as:

$$\begin{array}{l} F_{high}^{\prime }=F_{high}+d\left(\alpha ⊗F_{low}\right), \end{array}$$

where ⊗ denotes element-wise multiplication, *F* = *F*_*dilated*_ + *F*_*normal*_ for all layers,and *d*(·) represent 1 × 1 × 1 convolutions for aligning the dimensionality of that of the higher-level feature maps. α is formulated as the following:

$$\begin{array}{l} \alpha =\left[\alpha_{1},\alpha_{2},\ldots ,\alpha_{c}\right], \end{array}$$

$$\begin{array}{l} \alpha_{c}=\sigma \left(Z\left(B\left(Conv^{1\times 1\times 1}\left(AvgPool\left(F_{high}\right)\right)\right)\right)\right), \end{array}$$

where *Z* represents the rectified linear unit (ReLU) function, which provides non-linearity by setting negative values as zeros and keeping positive ones constant; *B* denotes the batch normalization (Ioffe and Szegedy, 2015) , which can accelerate and stabilize network training by standardizing each training batch; and σ denotes the sigmoid function for rescaling the attention coefficients to [0, 1].

### 2.4. Evaluation in a Multi-Site Data Set With Brain Pathology

**Figure 5:**
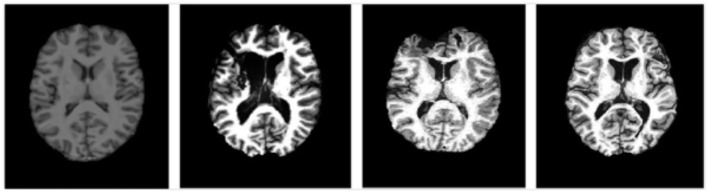
Representative images at similar anatomic levels from the source domain (a healthy subject, the leftmost) and target domains (3 TBI patients in the 3 rightmost frames). The slices were selected to highlight the lesion pathology and not the amygdala.

Amygdala segmentation strategies with CNN methods were also evaluated in a T1-weighted structural imaging study of children ages 9–18 years with severe traumatic brain injury (TBI) scanned 1–2 years after the injury. Twenty-one children (13F/8M) ages 9–18 years were scanned with T1w imaging at 13 sites with differing 3T MRI scanner systems, RF coils and pulse sequences. Among the TBI scans, 9 sites scanned one subject, 3 sites scanned two subjects and 1 site scanned six subjects. Representative images are shown in Figure 5. The data collection was approved by the Institutional Review Boards for each site and parental assent and informed consent was obtained for all subjects. Similar imaging protocols were employed across sites (3D T1w MP-RAGE (TI = 900 ms on Siemens and Philips) or BRAVO IR-fSPGR (TI = 450 ms on GE) with 1 mm isotropic spatial resolution (256 mm FOV with 256 × 256 matrix and 192 sagittal slices at 1 mm thick); however, there was variability between sites in terms of scanner manufacturers and models, RF coils, and pulse sequences, which affected spatial sensitivity, contrast, and image quality. Further, the severity, type and localization of injuries was extremely heterogeneous across sites. All these issues pose challenges on the applicability of CNNs, which typically do not generalize well to data whose distribution is different from that of the training data (Gibson et al., 2018a). Prior studies on multi-site generalized segmentation either retrains the model directly on multi-site data (Gibson et al., 2018a) or fine-tunes the domain-specific parameters (Karani et al., 2018) of the model, both requiring a few labeled target images from the new sites. In this study, we instead resort to pixel-level image adaptation, aiming to directly segment the full amygdala volumes from the multi-site images without the corresponding labels. We did not attempt to evaluate the segmentation of amygdala subregions for this multi-site study because manual labeling was deemed impractical for these data due to insufficient data quality for reliable identification.

As there was considerable site-to-site variability, we investigated the utility of a cycle-consistent generative adversarial network approach (CycleGAN) (Zhu et al., 2017) to harmonize the image contrast with the training data. CycleGAN has not been applied to multi-site data harmonization before, to the best of our knowledge. Specifically, the distribution of multi-site target data is transformed into source-like distribution while the appearance of the target images are preserved. In this way, a pre-trained segmentation model can be directly applied to the adapted target images without prior assumptions on scanner/protocol deviations. CycleGAN consists of two generators that learn two mappings, respectively, *G*_1_:*S* → *T* and *G*_2_:*T* → *S*, and two discriminators *D*_1_, *D*_2_ that distinguish the generated images from the real ones for each domain. In particular, we are interested in the generator *G*_2_ that transforms the target images into realistic source-like images, i.e., $G_{2}\left(x^{t}\right)=x^{t\to s}$. The distribution of the target and source images are aligned by applying adversarial losses (Goodfellow et al., 2014) where *G* tries to confuse *D* by producing realistic source-like images. Cycle-consistent losses (Zhu et al., 2017) computed by *l*_1_ distance are also applied to ensure that the generated target images are similar to the original ones. The transformed target images eventually obtained from the CycleGAN will be rendered as if they are drawn from the source domain, with the contents preserved. The total loss is defined as:

$$\begin{array}{l} L_{total}\left(G_{1},G_{2},D_{1},D_{2}\right)=L_{adv}\left(G_{1},D_{2}\right)+L_{adv}\left(G_{2},D_{1}\right)\\ \;+\lambda L_{cyc}\left(G_{1},G_{2}\right), \end{array}$$

where λ is used to modulate the strength of the cycle consistency.

### 2.5. Implementation Details

The proposed segmentation method was implemented in PyTorch, using one Titan Xp GPU for training. Categorical cross entropy was employed as the cost function, optimized via the Adam solver with an initial learning rate of 0.001, scheduled to decay as $lr=lr_{initial}*(1-\frac{iter_{n}}{total_{iter}}{\;)}^{power},$ where *power* was set to 0.9. Weights in each layer were initially drawn from a zero-based Gaussian distribution with standard deviation of $\sqrt{2/n_{i}},$ where *n* denotes the number of units in a kernel of the layer *l* (He et al., 2016). Bias were initialized at zero. Training was performed in batches of 14 image patches. In each iteration, 11 patches of size 59 × 59 × 59 were sampled from the whole brain and fed into the model. During inference, 105 × 105 × 105 patches were used. For comparison, training of the other deep learning based methods, i.e., *HighRes3DNet* (Li et al., 2017), *DeepMedic* (Kamnitsas et al., 2017) were implemented in Tensorflow (Gibson et al., 2018b) following their original settings in the respective papers, i.e., Dice loss (Milletari et al., 2016) was used in *HighRes3DNet* and categorical cross entropy in *DeepMedic*. An existing multi-atlas based method (Wang et al., 2014) was also evaluated for comparison in a leave-one-out fashion: 13 atlases were used for training and one atlas for evaluation. For all the deep learning based methods evaluated, a 7-fold cross validation was performed. In each fold, 10 subjects were used for training, 2 for validation and 2 for testing. The models were trained with a fixed number of epochs. The model parameters in the epoch that resulted in best performance (i.e., highest average dice) on the validation set were used to segment the test set. Performance of all methods on the test set was reported.

For multi-site MR image harmonization, we trained the CycleGAN on the coronal view of all the images from all domains. For the architecture choices, we followed the original settings: two convolutions with stride of 2, 9 residual blocks, two fractionally strided convolutions with stride $\frac{1}{2}$ are employed as the generator (Johnson et al., 2016), and 70 × 70 PatchGAN (Isola et al., 2017) is employed as the discriminator which aims to detect 70 × 70 image patches as real or fake. In total 3,304 slices from the source data and 5,900 slices from the TBI data are used for training. Each slice is randomly cropped to 128 × 128 before being fed into the CycleGAN. Data augmentation includes random rotation with angles of γ·90°, where γ ∈ [0, 1, 2, 3], and scaling with factors 0.8, 1, 1.2. For comparison only, we also conducted supervised training by training a model using the labeled TBI data in a 7-fold cross validation scheme, and the above-mentioned multi-atlas based method which was trained on the source data in a leave-one-out cross validation scheme and then directly applied to the TBI data. Results are summarized and analyzed in section 3.4.

### 2.6. Evaluation Metrics

The pair-wise similarity and discrepancy of our automatic (A) and manual segmentation (M) were evaluated using the commonly employed Dice Similarity Coefficient (DSC):

$$\begin{array}{l} DSC=\frac{2|A\cap M|}{\left|A|+|M\right|}, \end{array}$$

whose value ranges from zero to 1, where 1 indicates 100% with the ground truth, and 0 indicates no overlap. However, volumetric overlap measures are not sensitive to the contour of the segmentation output, while the latter is important in many medical applications such as disease diagnosis and treatment planning, as is also the case for the amygdala (Shenton et al., 2002; Tang et al., 2015; Yoon et al., 2016). Thus, we additionally consider a distance-based metric—the average symmetric surface distance (ASSD) (Geremia et al., 2011) in our evaluation. ASSD is defined as the average of distances between border voxels of our automatic segmentation output and those of manual segmentation output:

$$\begin{array}{l} ASSD\\ =\frac{\sum_{m\in B\left(M\right)}min_{a\in B\left(A\right)}||m-a||+\sum_{a\in B\left(A\right)}min_{m\in B\left(M\right)}||a-m||}{\left|B\left(M\right)|+|B\left(A\right)\right|}, \end{array}$$

where *B*(·) denotes the set containing all the voxels on the border. Zero value for this measure indicates a perfect segmentation.

## 3. Results

In this section, we present qualitative and quantitative results for our model and conduct ablation studies to demonstrate the effectiveness of each proposed component. We also compare the results of the proposed method with several state-of-the-art methods on the same dataset. Finally, we explore the feasibility of harmonizing the multi-site TBI data using CycleGAN and show the generalized capability of our method. Wilcoxon signed rank tests (two-sided) are used for performance comparison throughout the analysis.

### 3.1. Single-Branch vs. Dual-Branch

**Figure 6:**
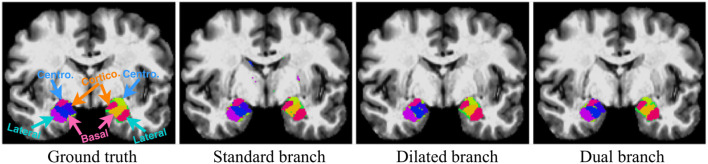
Qualitative segmentation examples show influences of each single branch on the final dual-branch model. The incorporation of larger context (Dilated branch) enables the final model to better localize the subregions, thus reducing false positives (the scattered misclassified background voxels, as seen on the Standard Branch result), while standard branch helps refine the appearance details of the final output.

Here we demonstrate the advantages of the dual-branch design by investigating the influences of each single branch. Experiments of using the dilated and standard branch separately are conducted in the same 7-fold cross validation scheme. Each branch is equipped with residual connections as in the original dual-branch setting. It can be observed in Table 1 that the *dilated* branch, which has a significantly larger receptive field, performs better on larger subregions (lateral, basal), while the *standard* branch with a smaller receptive field is better at segmenting smaller subregions, especially on the cortico-superficial subregions (*p* = 0.007). Additionally, the *dilated* branch yields significantly lower ASSD values than the *standard* branch on all subregions (*p* < 0.05). The *dual-branch* network inherits the merits of each single branch and achieves best overall accuracy in terms of both Dice and ASSD. Qualitative results of the compared models are shown in Figure 6.

**Table 1:** Dice overlap (columns 2–4) and ASSD (columns 5–7) performance of both single branch models and the dual-branch model.

**Subregions**		**Dice (%)**			**ASSD (mm)**	
	**Dilated**	**standard**	**Dual**	**Dilated**	**standard**	**Dual**
Lateral	80.6 (6.6)	77.9 (7.7)	**82.6 (5.0)**	0.70 (0.24)	2.66 (1.90)	**0.68 (0.31)**
Basal	76.6 (6.6)	75.9 (6.1)	**77.3 (6.0)**	**0.70 (0.15)**	1.10 (0.68)	0.71 (0.20)
Centromedial	73.7 (7.7)	**76.7 (5.2)**	75.4 (5.3)	**0.61 (0.16)**	1.00 (0.66)	0.61 (0.20)
Cortical-Superficial	71.7 (5.7)	72.2 (5.6)	**73.1 (5.6)**	0.96 (0.44)	1.94 (2.00)	**0.81 (0.33)**
Mean	75.6 (7.4)	75.7 (6.5)	**77.1 (6.4)**	0.74 (0.30)	1.67 (1.59)	**0.70 (0.27)**

*Subregions are listed in descending order by their volume-to-surface ratio. Highest are highlighted in bold and the second highest are underlined. The dual-branch model performance was either highest or second highest for all regions in terms of both Dice overlap or ASSD*.

### 3.2. Top-Down Attention-Guided Refinement Unit

**Figure 7:**
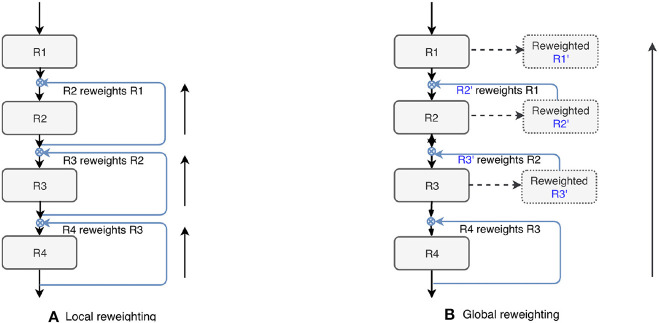
Two variants of the proposed top-down attention. RX denotes the residual blocks (residual connections are omitted here).

We also tested the effectiveness of the proposed top-down attention guided feature refinement scheme for further boosting the accuracy. Two variants were explored: “local reweighting” and “global reweighting,” as illustrated in Figure 7. These were compare with the SE blocks (Hu et al., 2018) that are also placed on the residual connections. Table 2 shows that the “local reweighting” scheme yields best overall Dice, especially on the cortical-superifical subregions (*p* < 0.05) which are the most challenging due to the smallest volume-to-surface ratio. Thus, we employ a “local reweighting” scheme for the attention module. Meanwhile, we can observe that the addition of either the “global reweighting” scheme or the SE blocks results in comparable or increased model complexity, while the results get slightly worse. This demonstrates that the improvements are indeed due to better feature refinement resulting from the locally top-down attention module, and not simply from the increased capacity of the model.

**Table 2:** Comparison for the Dice score (%) of the two variants and the SE blocks against the baseline (dual-branch model) and the percentage increase in model complexity.

**Subregions**	**Baseline**	***SE***	**Global**	**Local**
Lateral	82.6 (5.0)	81.2 (7.1)	**83.4 (5.1)**	82.8 (5.2)
Basal	77.3 (6.0)	76.9 (5.7)	77.2 (5.5)	**77.6 (5.3)**
Centromedial	75.4 (5.3)	74.5 (6.2)	76.3 (5.1)	**76.6 (5.6)**
Cortical-Superficial	73.1 (5.6)	71.7 (5.1)	72.5 (5.8)	**74.7 (5.6)**
Mean	77.1 (6.4)	76.1 (6.9)	77.4 (6.7)	**78.0 (6.1)**
Parameters (% increase)	0.795M (–)	0.811M (+2.0%)	0.808M (+1.6%)	0.808M (+1.6%)

*The largest value in each row is bold faced*.

### 3.3. Comparison With Other State-of-the-Art Methods

In order to demonstrate the advantage of the proposed method, we compared our method with some other popular publicly available segmentation methods including two deep learning models, *DeepMedic* and *HighRes3DNet*, and a multi-atlas based algorithm. *HighRes3DNet* is a state-of-the-art method in brain parcellation for 155 neuroanatomical structures (not including extremely small brain structures such as the subregions of the amygdala), and *DeepMedic* has shown excellent performance in lesion segmentation. Results (Table 3) show that our method exhibited superior performance in terms of both Dice and ASSD in this application. The differences in Dice with *DeepMedic* on the lateral (*p* = 0.04), basal (*p* = 0.03) and cortical-superficial (*p* < 0.005) subregions were significant. In particular, our method demonstrated substantial improvements for the cortical-superficial subregions thanks to the top-down attention guided refinement module. *DeepMedic* performed better ASSD on the basal subregions (*p* < 0.005) and our method were better at the cortical-superficial subregions (*p* < 0.03). Compared to multi-atlas, our method yielded significantly better Dice on the lateral, basal and cortical-superficial subregions (*p* < 0.05; *p* < 0.05; *p* < 10^−3^, respectively). There was no statistically significant differences on ASSD between our method and the multi-atlas based method.

**Table 3:** Mean and standard deviation of the Dice scores and ASSD for the proposed method, two other state-of-the-art deep learning based and a multi-atlas based segmentation methods evaluated on subregions.

**Methods**	**Lateral**	**Basal**	**Centromedial**	**Cortical-Superficial**	**Mean**
**DICE (%)**					
Multi-atlas	80.3 (7.0)	75.4 (6.1)	75.2 (6.4)	69.9 (5.7)	75.2 (7.3)
HighRes3DNet	68.1 (11.4)	69.3 (7.0)	25.3 (34.5)	65.8 (6.7)	57.1 (26.1)
DeepMedic	80.5 (7.5)	75.6 (6.5)	75.5 (5.3)	71.6 (4.2)	75.8 (6.7)
Dual (Ours)	82.6 (5.2)	77.3 (6.0)	75.4 (5.3)	73.1 (5.6)	77.1 (6.4)
Dual + Top-down Att (Ours)	**82.8 (5.0)**	**77.6 (5.3)**	**76.6 (5.7)**	**74.7 (5.4)**	**78.0 (6.1)**
**ASSD (mm)**					
Multi-atlas	**0.60 (0.20)**	0.73 (0.16)	**0.54 (0.12)**	0.75 (0.16)	**0.66 (0.18)**
HighRes3DNet	2.00 (1.26)	1.20 (0.43)	16.63 (12.20)	1.18 (0.51)	5.25 (8.96)
DeepMedic	1.13 (1.11)	**0.52 (0.36)**	0.76 (0.67)	1.37 (1.01)	0.94 (0.89)
Dual (Ours)	0.67 (0.31)	0.71 (0.20)	0.61 (0.20)	0.81 (0.33)	0.70 (0.27)
Dual + Top-down Att (Ours)	0.94 (1.30)	0.69 (0.15)	0.67 (0.42)	**0.73 (0.22)**	0.76 (0.70)

*“Dual” denotes the proposed segmentation model without the top-down attention guided feature refinement module. Highest are highlighted in bold and the second highest are underlined*.

### 3.4. Generalization on Multi-Site TBI Dataset

**Figure 8:**
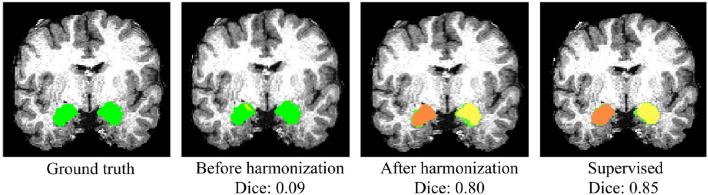
Qualitative results of whole-amygdala segmentation in a single TBI scan. Automated segmentation results are shown in orange and yellow, and the ground truth expert labeled segmentations are shown in green. The overlays show that the segmentation was very poor before CycleGAN harmonization (2nd column), but much improved after harmonization.

Whole-amygdala segmentation performance on the training data is reported in Table 4, which shows a roughly 90% overlap between the algorithm and ground truth. We investigated the generalization of the proposed method on a challenging multi-site TBI dataset by directly applying the trained whole-amygdala segmentation model to the TBI data. The results were evaluated relative to the “gold standard” defined by manual correction of Freesurfer amygdala segmentations by an expert (GK). Both Dice overlaps and ASSD were computed. For comparison only, we also conducted supervised training with TBI labels (corrected Freesurfer segmentations). As the objective was to evaluate the utility of CycleGAN for improving deep neural network (DNN)'s performance when testing on out-of-distribution data, the performance of competing CNN methods on the multi-site TBI data was not evaluated for these data. It is clear from Table 5 that a direct application of our trained model to the multi-site data demonstrated very poor performance, while after harmonization by CycleGAN, the trained model's performance on target data was significantly improved (*p* < 10^−6^). Supervised training yielded slightly higher performance. The multi-atlas based method, which is much less affected by the shift in data distribution, demonstrated similar performance to our method after harmonization, though the processing time is considerably longer. It should be noted that the segmentation performance for all the approaches was substantially lower than for the segmentation applied to the training data (Table 4). Qualitative results for one subject are shown in Figure 8.

**Table 4:** Dice overlap performance on the main training dataset using a leave-one-out approach (described in section 2.1).

***Amygdala***	**L. Amyg**	**R.Amyg**	**Mean**
Dice (%)	90.6 (2.1)	90.5 (2.1)	90.6 (1.9)

*This trained model is also applied to the harmonized TBI dataset*.

**Table 5:** Performance before and after harmonization using CycleGAN and supervised training using TBI labeled data, and a multi-atlas method.

**Settings**	**No harmonization**	**After harmonization**	**Supervised**	**Multi-atlas**
Dice (%)	42.4 (21.8)	75.5 (6.7)	76.0 (9.6)	75.0 (8.4)
ASSD (mm)	N/A	1.2 (0.7)	1.9 (1.7)	0.9 (2.9)

## 4. Discussion

In this study, we present a lightweight dual-branch residual FCNN with enhanced feature refinement to segment the subregions of the amygdala. Parallel branches with different dilation rates are used to process objects with different scales as well as extract more global contexts, and a top-down attention-guided refinement unit is proposed to guide the selection of lower level details for better feature refinement. We evaluated our method on MRI image data acquired from a cohort of adolescents. The results show that the proposed method achieved better performance as compared to several existing state-of-the-art segmentation methods. Meanwhile, our approach takes several seconds to segment the data of a subject, which is orders of magnitude faster than the multi-atlas based approach. This opens up the potential for real-time use during MRI acquisition, which would facilitate individualized functional, structural or spectroscopic imaging of small anatomical structures.

From the results of using each branch separately, we found that the performance on objects of different scales can be critically influenced by the receptive fields, and the proper receptive fields is correlated with the scale of objects. Our dual-branch design with different receptive fields thus flexibly adapt to subregions of different scales. Furthermore, although the standard branch with a small receptive field is prone to spatial inconsistencies due to local similarities, dilated branch remedies this effect by incorporating more global contextual information via dilated convolutions. The significantly lower ASSD values it yields suggest that dilated branch is especially effective in reducing such false positives, indicating its strong localization ability for ROIs and boundaries. This suggests that each branch provides complementary information toward the solution of the segmentation problem. Benefiting from both branches, the final model obtained substantially more accurate segmentation results both volumetrically and morphologically.

Besides the lightweight dual-branch backbone, we also explore the idea of multi-scale fusion and enhance it with a top-down attention-guided refinement unit. An important design choice for the proposed refinement unit is the strategy to use more local or global high-level information as the guide. The results indicate that the local refinement scheme may be more suitable and it is especially advantageous in small and challenging subregions (cortical-superficial). This is consistent with our hypothesis that smaller objects tend to benefit more from feature reuse. Interestingly, the comparison with SE blocks suggest that SE blocks inhibit rather than emphasize the ROIs in this application, as also found in Roy et al. (2018). This may due to the small size of the features of the ROIs whose contribution to the whole feature maps are less significant compared with other features of the same level and are thus suppressed. We therefore speculate that the top-down design, which utilizes higher semantic and categorical information as priors to determine the importance the lower-level features, may alleviate this problem and thus may be more suitable for segmentation tasks of small objects.

In comparisons with two other state-of-the-art deep learning models, our method shows superior performance in terms of both Dice overlap and ASSD. Notably, all evaluated models contain comparable parameters and therefore comparable capacities, while they vary in their topological structures. HighRes3DNet consists of consecutive 20 dilated residual convolutional layers with progressively enlarged dilation rates. It shares many key components with the backbone of our model such as the dilated residual convolutions, but has them connected in series only while ours also in parallel. Such serial connections result in an overly large receptive field (87 × 87 × 87) which causes severe class imbalance in segmenting small and compact subregions that cannot seem to be well resolved by using Dice loss, as indicated in Table 3. This also demonstrates the benefit of having an another branch that maintains a small receptive field in our model design. DeepMedic consists of two independent branches with the second branch processing a low-resolution version of the inputs. Compared with HighRes3DNet, the architecture of DeepMedic is flexible enough to process input segments with smaller spatial sizes, which can inherently balance the distribution of different classes. DeepMedic also exploits multi-scale learning scheme, but the responses of two branches are not fused until the very end of the network. In contrast, our model encourages interactions of multi-resolution features both in parallel and in series. This could explain the improved performance of even our dual-branch model with respect to DeepMedic, though they have the same model complexity.

Finally, we evaluate the generalizability of our method on a multi-site TBI dataset by first pre-training the model on the main dataset and then directly applying it to the TBI data. In order to address domain shifts, we explore the feasibility of harmonizing the multi-site data using CycleGAN, which is shown to be effective and nearly closes the gap to supervised training (i.e., training with TBI labels) in this application. Comparing the Dice overlap performance of the supervised training on the main dataset and the TBI dataset, the accuracy drop on TBI data (90% to 76%) may be attributed to high variations due to heterogeneous scanning methods and anatomical injuries. Thus, larger labeled datasets are desired for better training for TBI studies, which however are often not feasible in medical imaging where expert-defined labels are often rare. Our results show that after a decent data harmonization by CycleGAN, using a single small set (*N*≈14) of high-quality labeled data (even though they are healthy subjects) can approximate the accuracy of directly training with a few (*N*≈21) TBI labeled data. This suggests that our solution makes it possible to reuse labels from different domains and thus alleviate the burdens for labeling. Another important advantage is that knowledge of sources of biases from scanners/protocols are not required for harmonization using CycleGAN. A limitation, however, is that CycleGAN only adapts images at pixel-level while feature spaces should ideally be aligned as well for better domain adaptation, which we leave for future works. Another limitation with this study was that only the whole amygdala segmentations were evaluated because the raw T1-weighted images were not of sufficient quality for expert manual labeling of the subregions.

## 5. Conclusion

In this study, we presented a novel dual-branch dilated residual FCNN with enhanced feature fusion via a top-down attention-guided refinement unit to segment the subregions of the amygdala with high accuracy. Each branch with a different receptive field demonstrated specialized ability of processing objects of the corresponding scale, thus providing complementary information. Also, we found that the proposed attention-guided feature refinement module may be more suitable than the SE blocks in segmenting small structures due to the top-down design. The proposed model showed superior performance compared with two state-of-the-art deep learning methods. Our method also shows decent generalizability on a challenging multi-site TBI dataset without needing to be re-trained, after harmonizing the TBI data using a CycleGAN. We believe that our findings and the model design could provide insights especially on generalized segmentation of small objects, which are relatively under-studied, and the high efficiency of our technique will potentially benefit real-time use in clinical practices.

## Data Availability Statement

The datasets generated/analyzed for this study can be found in https://www.nitrc.org/projects/amyg_autoseg.

## Ethics Statement

The studies involving human participants were reviewed and approved by University of Wisconsin - Madison Health Sciences IRB. Written informed consent to participate in this study was provided by the participant, or the participants' legal guardian/next of kin).

## Author Contributions

YL, GZ, NA, MS, and AA contributed to the conception and design of the work. BN, PF, and AA contributed to the acquisition of the data for the study. YL, BN, MS, GK, and GZ contributed to the analyses and interpretation of the data. YL wrote the first draft of the manuscript. BN, MS, and AA provided significant contributions to the writing. All authors contributed to manuscript revision, read and approved the submitted version.

## Conflict of Interest

AA is Co-owner of Thervoyant, Inc. The remaining authors declare that the research was conducted in the absence of any commercial or financial relationships that could be construed as a potential conflict of interest.
